# Effectiveness and Implementation of Physical Activity Interventions for Rural Breast Cancer Survivors: A Systematic Review

**DOI:** 10.7759/cureus.106172

**Published:** 2026-03-30

**Authors:** Ryuichi Ohta, Taichi Fujimori, Kaoru Tanaka, Hidetoshi Hayashi

**Affiliations:** 1 Department of Community Care, Unnan City Hospital, Unnan, JPN; 2 Department of Internal Medicine, Shimane University, Shimane, JPN; 3 Department of Medical Oncology, Kindai University Faculty of Medicine, Sakai, JPN

**Keywords:** breast neoplasms, cancer survivors, exercise therapy, motor activity, quality of life, rehabilitation, rural population, telemedicine

## Abstract

Physical activity is recommended for breast cancer survivors to improve physical function, reduce treatment-related symptoms, and enhance quality of life. However, survivors living in rural areas often face barriers to participating in exercise programs due to geographic isolation, limited access to rehabilitation services, and fewer community resources. Although various exercise interventions have been developed for cancer survivors, evidence regarding their effectiveness and implementation in rural populations remains limited. This systematic review examined the effectiveness and feasibility of physical activity interventions for breast cancer survivors living in rural or non-urban settings. A systematic review was conducted in accordance with a predefined protocol. Electronic databases were searched to identify studies evaluating structured physical activity or rehabilitation programs for adult breast cancer survivors residing in rural areas. Eligible study designs included randomized controlled trials, quasi-experimental studies, and observational studies. Two reviewers independently screened studies, extracted data, and assessed methodological quality. Outcomes included physical function, physical activity levels, health-related quality of life, fatigue, adherence, and feasibility. Findings were synthesized narratively due to heterogeneity in study designs and outcome measures. Seven studies met the inclusion criteria. Interventions included telephone-based counseling, tele-exercise programs, lifestyle interventions, and community-based physical activity initiatives. Most studies reported improvements in physical activity participation, physical performance, lifestyle behaviors, or quality of life. Several interventions demonstrated measurable benefits, including increased physical activity levels and improvements in functional outcomes. Remote delivery approaches, such as telephone or telehealth programs, were frequently used to address geographic barriers. Risk-of-bias assessment indicated generally low to moderate methodological quality across the included studies. Physical activity interventions appear to be feasible and beneficial for breast cancer survivors living in rural areas. Remote and community-based delivery approaches may help overcome access barriers and support participation in exercise programs. Future research should explore sustainable, community-engaged models that promote long-term physical activity and improve quality of life among rural cancer survivors.

## Introduction and background

Breast cancer is one of the most diagnosed malignancies worldwide, and the number of survivors continues to increase due to advances in early detection and treatment outcomes [1]. As survival improves, a growing population of breast cancer survivors experiences long-term physical and psychological consequences of cancer and its treatment. Common survivorship issues include cancer-related fatigue, decreased physical function, reduced muscle strength, upper limb dysfunction, and impaired health-related quality of life (HRQoL) [2].

Physical activity and exercise-based rehabilitation have been widely recognized as effective interventions to address these challenges. Numerous randomized controlled trials (RCTs) and meta-analyses have demonstrated that structured physical activity programs can improve physical function, reduce cancer-related fatigue, and enhance quality of life among breast cancer survivors [3,4]. Consequently, exercise interventions are increasingly recommended as part of comprehensive survivorship care.

However, most studies evaluating exercise-based rehabilitation have been conducted in urban or resource-rich settings where access to rehabilitation professionals and healthcare facilities is readily available. In contrast, breast cancer survivors living in rural areas often face significant barriers to accessing such programs. These barriers include long travel times to medical facilities, limited availability of rehabilitation specialists, transportation difficulties, and disparities in digital infrastructure [5-7]. Furthermore, rural populations often have a higher proportion of older adults with lower physiological reserve, making the sustainability and adherence to physical activity interventions a critical concern [8].

To address these challenges, alternative intervention models, such as home-based exercise programs and tele-rehabilitation approaches, have recently been developed. These approaches aim to provide accessible, scalable rehabilitation programs across geographically dispersed regions while reducing travel burdens and addressing healthcare resource limitations [9,10]. Nevertheless, despite the increasing number of intervention studies targeting rural populations, the effectiveness, sustainability, and implementation characteristics of these interventions remain insufficiently synthesized.

From the perspective of rehabilitation implementation in community healthcare settings, it is essential to evaluate not only the clinical effectiveness of physical activity interventions but also their adherence, safety, and delivery models. Maintaining physical function in older breast cancer survivors is closely linked to independence and participation in community life. Therefore, identifying effective and sustainable intervention strategies tailored to rural environments is an important research priority.

Therefore, this systematic review aims to comprehensively evaluate the effectiveness and sustainability of physical activity interventions for breast cancer survivors living in rural areas. Specifically, this study examines the effects of these interventions on physical function as the primary outcome and evaluates secondary outcomes, including HRQoL, cancer-related fatigue, upper limb function, muscle strength, and physical activity levels. In addition, intervention adherence, dropout rates, safety, and differences in intervention delivery methods are assessed to identify feasible rehabilitation strategies for rural healthcare settings.

## Review

Study design

This study was designed as a systematic review and meta-analysis to examine the effectiveness and long-term sustainability of physical activity interventions among breast cancer survivors residing in rural settings. The review process was conducted following the recommendations outlined in the Preferred Reporting Items for Systematic Reviews and Meta-Analyses (PRISMA) guidelines [11].

Protocol registration

The review protocol was prospectively registered in the International Prospective Register of Systematic Reviews (PROSPERO). The registration number is CRD420261324114.

Search strategy

A comprehensive literature search was conducted in the electronic databases PubMed/MEDLINE, Embase, and Web of Science. Studies published from database inception through January 2026 were considered eligible. The search strategy was developed using a combination of Medical Subject Headings (MeSH) and relevant free-text keywords associated with breast cancer, physical activity, and rural populations. Representative search terms included “breast cancer,” “breast cancer survivors,” “physical activity,” “exercise,” “rehabilitation,” “rural,” “non-urban,” “tele-rehabilitation,” and “home-based exercise.” These terms were combined using Boolean operators (AND/OR) to optimize the retrieval of relevant studies. The search was restricted to articles published in English to ensure consistency in data extraction and interpretation. Detailed search strategies for each database are provided in the Appendix. Gray literature, conference abstracts, and unpublished studies were excluded because they had not undergone peer review. Additionally, the reference lists of all included studies were manually reviewed to identify further relevant publications.

Study selection

Studies were included if they satisfied predefined eligibility criteria based on participants, interventions, comparators, outcomes, and study design. Eligible participants were adults (≥18 years) diagnosed with breast cancer who lived in rural areas or were analyzed as a rural subgroup within a broader study population.

The interventions of interest were structured physical activity or rehabilitation programs aimed at improving physical functioning in breast cancer survivors. These programs included aerobic exercise, resistance training, upper extremity functional training, home-based exercise interventions, tele-rehabilitation or telehealth-based programs, and community-based rehabilitation initiatives. Comparator groups included usual care, minimal intervention, waiting-list controls, or pre-post assessments in single-arm intervention studies. The primary outcome was physical function, measured using objective indicators such as the six-minute walk test (6MWT), upper limb functional performance, range of motion (ROM), and muscle strength assessments. Secondary outcomes included HRQoL, cancer-related fatigue, levels of physical activity, adherence to interventions, dropout rates, and reported adverse events.

Eligible study designs included RCTs, non-randomized comparative studies, cohort studies, and quasi-experimental designs. Case reports, narrative reviews, editorials, and conference abstracts were excluded. Two reviewers independently screened the titles and abstracts of all retrieved records to identify potentially relevant studies. Full-text articles of the selected studies were subsequently assessed according to the predefined eligibility criteria. Discrepancies between reviewers were resolved through discussion, and when consensus could not be reached, a third reviewer was consulted to make the final decision.

Data extraction and synthesis

Data extraction was conducted independently by two reviewers using a predefined and standardized data collection form. The extracted information included general study characteristics (first author, year of publication, and country), study design, and participant details such as sample size, age, and cancer stage. Detailed information regarding the interventions was also collected, including the type of intervention, delivery format, and duration. Reported outcome measures were extracted, focusing on indicators of physical function, HRQoL, cancer-related fatigue, and levels of physical activity. In addition, data on intervention adherence, dropout rates, and any reported adverse events were recorded to evaluate the feasibility and safety of the interventions. The extracted data were summarized descriptively to provide an overview of study characteristics and intervention strategies. Interventions were further grouped according to their delivery modality, such as home-based programs, tele-rehabilitation approaches, and community-based rehabilitation interventions.

Risk-of-bias assessment

The methodological quality of the included studies was independently evaluated by two reviewers. RCTs were assessed using the Cochrane Risk of Bias 2 (RoB 2) tool, which examines potential sources of bias related to the randomization process, deviations from intended interventions, missing outcome data, outcome measurement, and selective reporting of results [12]. Non-randomized studies were appraised using the Risk Of Bias In Non-randomized Studies of Interventions (ROBINS-I) tool, which evaluates possible bias arising from confounding factors, participant selection, classification of interventions, deviations from intended interventions, incomplete data, outcome assessment, and selective reporting [13]. Discrepancies between reviewers were addressed through discussion until agreement was achieved. The findings of the risk-of-bias assessment were summarized descriptively and presented in tabular format.

Statistical analysis

When comparable data were available across studies, a meta-analysis was performed using a random-effects model to account for potential variability between studies. Continuous outcomes were summarized as standardized mean differences (SMDs) with corresponding 95% confidence intervals (CIs). Statistical heterogeneity was assessed using the I² statistic. The degree of heterogeneity was interpreted as low (<25%), moderate (25-50%), or substantial (>50%). If substantial heterogeneity was observed, subgroup analyses were planned according to intervention type, delivery modality, and participant age categories. Potential publication bias was evaluated through visual inspection of funnel plots when at least 10 studies were available for a specific outcome. Statistical analyses were conducted using EZR (Easy R), a graphical user interface for R developed for biostatistical analysis [14].

Results

Study Selection

**Figure 1:**
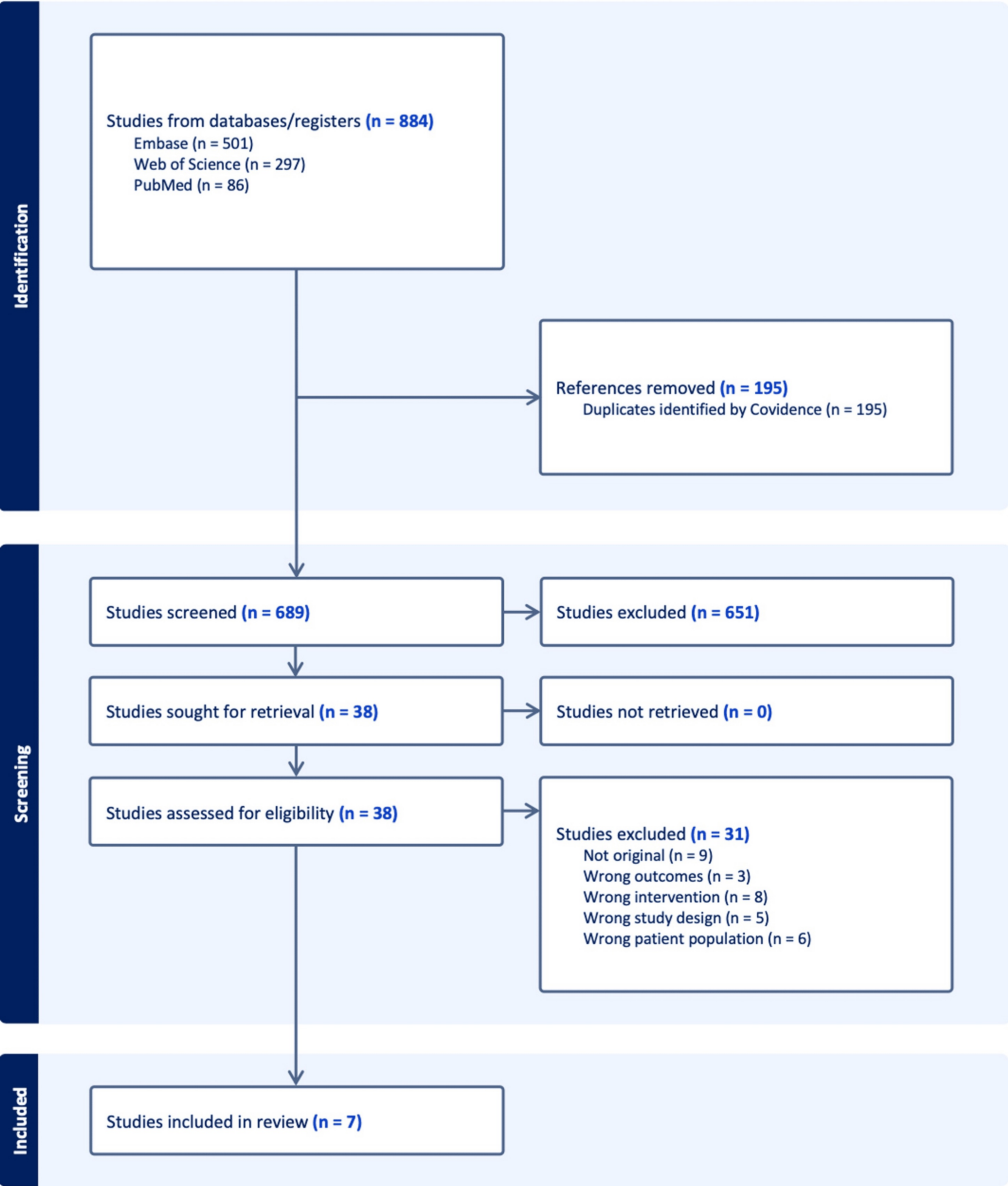
Selection flow

The study selection process is summarized in the PRISMA flow diagram. A total of 884 records were initially identified through database searches, including Embase (n = 501), Web of Science (n = 297), and PubMed (n = 86). After removing 195 duplicate records identified using Covidence, 689 studies remained for title and abstract screening. During the screening stage, 651 records were excluded based on titles and abstracts. The remaining 38 studies were retrieved and assessed for full-text eligibility. Among these, 31 studies were excluded for the following reasons: not original research (n = 9), wrong outcomes (n = 3), wrong intervention (n = 8), wrong study design (n = 5), and wrong patient population (n = 6). Ultimately, seven studies met the predefined inclusion criteria and were included in the final systematic review. No studies were excluded due to the inability to retrieve the full text. The detailed selection process is presented in the PRISMA flow diagram (Figure 1).

Characteristics of the Included Articles

A total of seven studies investigating physical activity or lifestyle interventions for breast cancer survivors residing in rural or non-urban settings were included in this review. The included studies were conducted in three countries, with the majority in the United States, and additional studies in Australia and Greece. The study designs included RCTs, quasi-experimental studies, and prospective intervention studies. Among the seven studies, several were randomized trials evaluating structured physical activity or lifestyle interventions, whereas others employed single-arm intervention designs to assess feasibility and preliminary effectiveness.

Sample sizes varied considerably across studies, ranging from 13 to 210 participants, reflecting both pilot studies and larger intervention trials. Participants were adult breast cancer survivors who had completed primary cancer treatment or were undergoing survivorship care.

The types of interventions also varied across studies. Most interventions focused on structured physical activity or lifestyle modification programs, including aerobic exercise, resistance training, or combined exercise programs. Several studies delivered interventions via remote or distance-based approaches, such as telephone counseling or tele-exercise programs, specifically designed to overcome geographic barriers faced by rural populations. Other studies implemented community- or home-based exercise programs to improve accessibility and long-term adherence.

Intervention durations ranged from eight weeks to 24 months, with many programs incorporating behavioral support components such as goal setting, self-monitoring, and peer support to enhance adherence to physical activity.

Across the included studies, the most commonly evaluated outcomes were physical function, quality of life, fatigue, and physical activity levels. Some studies also assessed additional outcomes such as weight loss, cognitive function, or biomarker changes associated with exercise interventions.

Overall, the included studies demonstrated substantial variability in intervention design, delivery methods, and outcome measures. Nevertheless, most interventions aimed to improve physical function and survivorship outcomes through accessible physical activity programs tailored to rural breast cancer survivors. The main characteristics of the included studies are summarized in Table 1.

**Table 1:** Characteristics of the included studies evaluating physical activity interventions for breast cancer survivors in rural or non-urban settings This table summarizes the characteristics of the studies included in this systematic review. Information presented includes country, study design, participant characteristics, intervention type, delivery method, duration, and reported outcomes. Primary outcomes mainly included weight loss, physical activity participation,  physical performance, or neurocognitive performance. Secondary outcomes commonly included quality of life, fatigue, biomarkers, psychological distress, and cost-effectiveness. Adherence or retention rates were reported to assess feasibility and participant engagement. BC, breast cancer; PA, physical activity; QoL, quality of life; RCT, randomized controlled trial; IGF-1, insulin-like growth factor 1.

Study	Country	Study Design	Participants	Intervention	Delivery Method	Duration	Primary Outcomes	Secondary Outcomes	Adherence/Retention	Key Findings
Befort et al., 2012 [15]	USA	One-arm intervention	34 rural breast cancer survivors	Lifestyle weight loss program (diet + physical activity)	Group conference call	6 months	Weight loss	Physical activity, diet, biomarkers, QoL	91% attended ≥75% sessions	Mean weight loss 13.9%; significant improvements in physical activity and QoL
Eakin et al., 2012 [16]	Australia	Randomized controlled trial	143 non-urban breast cancer patients	Aerobic + resistance exercise program	Telephone coaching	8 months	Physical activity participation	QoL, fatigue, upper body function	96% retention	Telephone exercise intervention improved activity levels
Befort et al., 2014 [17]	USA	Randomized controlled trial	210 rural breast cancer survivors	Behavioral weight loss + maintenance support	Group phone counseling vs newsletter	24 months	Weight loss maintenance	QoL, biomarkers, cost-effectiveness	High retention	Phone counseling improved long-term weight maintenance
Befort et al., 2016 [18]	USA	Randomized controlled trial	Rural breast cancer survivors	Lifestyle weight management program	Phone group sessions	18 months	Weight regain prevention	Cost effectiveness	High adherence	Phone-based intervention superior to newsletter
Myers et al., 2022 [19]	USA	Randomized pilot study	Breast cancer survivors	Exercise + game-based cognitive training	Hybrid supervised training	16 weeks	Neurocognitive performance	IGF-1 biomarker	Feasible	Increased IGF-1 levels associated with improved cognitive function
Andrioti et al., 2023 [20]	Greece	Longitudinal intervention	13 breast cancer survivors	Tele-exercise program	Home-based remote supervision	8 weeks	Physical performance	QoL, fatigue, anxiety	Good adherence	Improved fitness, fatigue, and QoL
Mama et al., 2025 [21]	USA	Feasibility RCT protocol	Rural breast cancer survivors	Mind Your BEAT physical activity program	Community-based intervention	Ongoing	Feasibility outcomes	Psychosocial distress, physical activity	Ongoing	Community-adapted PA program targeting rural survivors

Intervention Characteristics

Across the seven included studies, intervention strategies primarily focused on promoting physical activity or lifestyle modification among breast cancer survivors residing in rural or non-urban settings. Most interventions incorporated structured physical activity programs, including aerobic exercise, resistance training, or a combination of exercise modalities. Several studies integrated behavioral components, such as goal setting, self-monitoring, motivational support, or peer support, to enhance adherence to physical activity interventions.

Regarding delivery methods, many interventions were designed to address geographic barriers in rural settings by utilizing remote delivery approaches, such as telephone counseling, group conference calls, or tele-exercise platforms. These remote intervention strategies were intended to increase access for participants with limited access to rehabilitation services due to long travel distances or shortages of healthcare professionals in rural regions. Other studies implemented community-based or hybrid intervention models that combined supervised exercise sessions with home-based activities.

The duration of the interventions varied considerably across studies, ranging from eight weeks to 24 months. Several programs involved regular coaching sessions or supervised exercise training conducted weekly or multiple times per week. Behavioral support strategies were frequently incorporated to facilitate long-term engagement in physical activity.

Overall, despite variations in intervention design and delivery methods, the included studies consistently aimed to improve physical function and survivorship outcomes by providing accessible and sustainable physical activity programs tailored to rural breast cancer survivors. The characteristics of the interventions included in the reviewed studies are summarized in Table 2.

**Table 2:** Characteristics of physical activity interventions included in the review This table summarizes the characteristics of the exercise interventions evaluated in the included studies. Information presented includes the type of intervention, exercise components, delivery method, level of supervision, intervention frequency and duration, and behavioral support strategies used to promote adherence. Most interventions incorporated aerobic exercise with additional lifestyle or resistance-training components and were delivered remotely via telephone or tele-exercise to address access barriers in rural settings. PA, physical activity.

Study	Intervention Type	Exercise Components	Delivery Method	Supervision	Frequency	Duration	Behavioral Support
Befort et al., 2012 [15]	Lifestyle weight loss + physical activity	Aerobic exercise + diet modification	Group conference call	Remote supervision	Weekly sessions	6 months	Goal setting, self-monitoring, peer support
Eakin et al., 2012 [16]	Exercise intervention	Aerobic + resistance training	Telephone coaching	Remote supervision	Regular coaching	8 months	Motivational support
Befort et al., 2014 [17]	Behavioral weight loss intervention	Aerobic exercise + lifestyle modification	Telephone counseling	Remote supervision	Regular coaching sessions	24 months	Behavioral counseling
Befort et al., 2016 [18]	Weight management program	Physical activity + diet	Phone-based group sessions	Remote supervision	Regular sessions	18 months	Behavioral support
Myers et al., 2022 [19]	Tele-exercise program	Aerobic + resistance training	Home-based tele-exercise	Remote supervision	2 sessions/week	8 weeks	Online guidance
Andrioti et al., 2023 [20]	Exercise + cognitive training	Aerobic exercise + game-based cognitive tasks	Hybrid supervised training	Partially supervised	Multiple sessions per week	16 weeks	Performance feedback
Mama et al., 2025 [21]	Community physical activity program	General physical activity promotion	Community-based intervention	Community support	Community sessions	Ongoing	Community engagement

Outcome Characteristics

Among the included studies, three studies conducted by Befort et al. (2012, 2014, 2016) primarily evaluated lifestyle and weight management interventions delivered via telephone-based counseling [15,17,18]. Befort et al. (2012) [15] reported significant weight loss and increases in physical activity levels following a six-month group-based intervention. In their subsequent randomized trial, Befort et al. (2014) [17] demonstrated improvements in weight maintenance and lifestyle behaviors over a longer follow-up period. A later study by Befort et al. (2016) [18] further confirmed that telephone-based interventions could sustain lifestyle improvements among rural breast cancer survivors.

Exercise-focused interventions were evaluated in several studies. Eakin et al. (2012) reported that a telephone-delivered physical activity intervention significantly increased physical activity participation and reduced cancer-related fatigue [16]. Similarly, Andrioti et al. (2023) demonstrated improvements in physical performance, quality of life, and fatigue following a tele-exercise intervention [20].

Other studies evaluated broader survivorship outcomes. For example, Myers et al. (2022) investigated a combined exercise and game-based cognitive training program and observed improvements in cognitive performance and associated biomarker changes [19]. Finally, Mama et al. (2025) reported the feasibility of a community-based physical activity program designed for rural breast cancer survivors [21]. The outcomes evaluated in the included studies are summarized in Table 3.

**Table 3:** Outcomes of physical activity interventions among rural breast cancer survivors This table summarizes the outcome measures and main findings of the exercise interventions evaluated in the included studies. Outcomes commonly assessed across studies included physical activity participation, weight management, physical performance, cognitive function, fatigue, and health-related quality of life. Overall, the interventions demonstrated improvements in physical activity levels, functional outcomes, and quality of life, while some studies also reported benefits in weight control and cognitive performance. IGF-1, insulin-like growth factor 1.

Study	Intervention	Outcome Measures	Main Findings
Befort et al., 2012 [15]	Telephone lifestyle weight-loss program	Weight, physical activity, quality of life	Significant weight loss and increased physical activity after the intervention
Eakin et al., 2012 [16]	Telephone-delivered exercise intervention	Physical activity participation, fatigue, quality of life	Increased physical activity participation and reduced cancer‑related fatigue
Befort et al., 2014 [17]	Behavioral weight-loss intervention (telephone counseling)	Weight maintenance, quality of life	Improved long‑term weight control and lifestyle behavior
Befort et al., 2016 [18]	Lifestyle intervention for weight management	Physical activity behavior, weight management	Sustained improvements in physical activity and lifestyle behavior
Myers et al., 2022 [19]	Exercise plus game-based cognitive training	Cognitive performance, IGF‑1 biomarker	Improved cognitive performance and exercise capacity
Andrioti et al., 2023 [20]	Tele-exercise program	Physical performance, fatigue, quality of life	Improved physical performance, reduced fatigue, and improved quality of life
Mama et al., 2025 [21]	Community-based physical activity program	Feasibility outcomes, physical activity participation	Demonstrated feasibility of a community‑based physical activity program

Quantitative Summary of Outcomes

Among the included studies, three investigations conducted by Befort et al. (2012, 2014, and 2016) primarily evaluated lifestyle interventions targeting weight control and physical activity among rural breast cancer survivors [15,17,18]. In the six-month telephone-based intervention reported by Befort et al. (2012), participants experienced a mean weight reduction of 12.5 ± 5.8 kg and a substantial increase in physical activity of 1235 ± 832 kcal per week [15]. Subsequent trials by Befort et al. (2014) and Befort et al. (2016) further demonstrated improvements in long-term weight management and lifestyle behaviors [17,18].

In the RCT conducted by Eakin et al. (2012), participants receiving a telephone-delivered exercise intervention showed significantly higher participation in physical activity compared with controls [16]. Similarly, Andrioti et al. (2023) reported improvements in physical performance and fatigue following a tele-exercise intervention delivered remotely [20].

In addition, Myers et al. (2022) investigated an intervention combining exercise with game-based cognitive training and observed improvements in cognitive performance accompanied by increases in IGF-1 levels [19]. Finally, Mama et al. (2025) evaluated the feasibility of a community-based physical activity program and reported that the intervention was feasible and acceptable for rural breast cancer survivors [21]. The quantitative outcomes of all seven included studies are summarized in Table 4.

**Table 4:** Quantitative outcomes of physical activity interventions among rural breast cancer survivors This table summarizes the primary outcomes and key quantitative findings reported in the included studies. The outcomes primarily focused on weight change, physical activity participation, physical performance, cognitive function, and feasibility of interventions. Several studies reported measurable improvements in physical activity levels and lifestyle behaviors, while others demonstrated benefits in weight management or cognitive performance following exercise-based interventions. IGF-1, insulin-like growth factor 1; PA, physical activity.

Study	Intervention	Primary Outcome	Key Quantitative Result
Befort et al., 2012 [15]	Telephone lifestyle intervention (diet + physical activity)	Weight change/Physical activity	Weight −12.5 ± 5.8 kg; physical activity +1235 ± 832 kcal/week
Eakin et al., 2012 [16]	Telephone-delivered exercise intervention	Physical activity participation	Significant increase in physical activity participation
Befort et al., 2014 [17]	Behavioral weight-loss intervention	Weight maintenance	Improved long-term weight maintenance (reported improvement)
Befort et al., 2016 [18]	Lifestyle weight management program	Lifestyle behavior/physical activity	Improved physical activity behavior (reported improvement)
Myers et al., 2022 [19]	Exercise plus game-based cognitive training	Cognitive function	Increase in IGF‑1 and improved cognitive performance
Andrioti et al., 2023 [20]	Tele-exercise program	Physical performance	Improved physical performance measures
Mama et al., 2025 [21]	Community-based physical activity program	Feasibility of intervention	Program feasibility demonstrated

Quality Assessment Results

RCTs conducted by Befort et al. (2014) [17], Befort et al. (2016) [18], and Eakin et al. (2012) [16] generally demonstrated low risk of bias in randomization and outcome reporting domains, although blinding of participants was not feasible due to the behavioral nature of the interventions.

Single-arm or non-randomized studies, including Befort et al. (2012) [15] and Andrioti et al. (2023) [20], were associated with a higher risk of bias primarily due to the absence of control groups. Studies focusing on feasibility, such as Mama et al. (2025), also showed methodological limitations, including incomplete outcome reporting [21]. Overall, the methodological quality across the included studies ranged from low to moderate, with the most common limitations related to lack of blinding and small sample sizes. The methodological quality of all seven included studies was assessed using a structured risk-of-bias framework, and the results are presented in Table 5.

**Table 5:** Risk-of-bias assessment of the included studies This table presents the risk-of-bias assessment for the included studies across key methodological domains: randomization, allocation concealment, blinding of participants and personnel, incomplete outcome data, and selective reporting. Overall risk of bias was determined by evaluating these domains. Most randomized trials showed low risk in randomization and reporting domains, while blinding was commonly rated as high risk due to the behavioral nature of exercise interventions. RCT, randomized controlled trial.

Study	Randomization	Allocation Concealment	Blinding of Participants/Personnel	Incomplete Outcome Data	Selective Reporting	Overall Risk
Befort et al., 2012 [15]	High risk (single-arm study)	Not applicable	High risk	Low risk	Low risk	Moderate
Eakin et al., 2012 [16]	Low risk	Some concerns	High risk	Low risk	Low risk	Moderate
Befort et al., 2014 [17]	Low risk	Low risk	High risk	Low risk	Low risk	Low
Befort et al., 2016 [18]	Low risk	Low risk	High risk	Low risk	Low risk	Low
Myers et al., 2022 [19]	Some concerns	Some concerns	High risk	Some concerns	Some concerns	Moderate
Andrioti et al., 2023 [20]	High risk (non-randomized)	High risk	High risk	Some concerns	Some concerns	High
Mama et al., 2025 [21]	Some concerns	Some concerns	High risk	Some concerns	Some concerns	Moderate

Discussion

Summary of the Study

This systematic review examined the effectiveness and feasibility of physical activity interventions for breast cancer survivors living in rural or non-urban areas. Across the seven included studies, physical activity interventions were delivered through a variety of approaches, including telephone-based counseling, tele-exercise programs, and community-based physical activity initiatives.

Overall, the included studies consistently reported improvements in physical activity participation, functional performance, quality of life, or lifestyle behaviors following exercise-related interventions. Several studies demonstrated measurable changes in health-related outcomes. For example, Befort et al. [15] reported substantial weight reduction and increased physical activity following a telephone-based lifestyle intervention, while Eakin et al. [16] demonstrated increased physical activity participation through a telephone-delivered exercise program. Similarly, tele-exercise interventions reported improvements in physical performance and fatigue outcomes.

These findings suggest that physical activity interventions, particularly those designed to overcome geographic barriers, can contribute to improving health outcomes among rural breast cancer survivors. Importantly, many interventions introduced exercise programs gradually, allowing participants to increase activity levels progressively in line with their physical condition and treatment-related limitations. Such gradual implementation appears to be an important strategy for promoting safe and sustainable exercise participation among cancer survivors.

Comparison With Other Studies

The findings of this review are consistent with previous research demonstrating the benefits of exercise interventions in cancer survivorship care [22-24]. Numerous studies conducted in urban or hospital-based settings have shown that structured exercise programs can improve physical function, reduce fatigue, and enhance quality of life among breast cancer survivors [25,26].

However, most previous intervention studies have focused on programs delivered within clinical or rehabilitation facilities [27]. In contrast, the studies included in this review highlight the importance of alternative delivery models, such as telephone-based interventions, tele-exercise programs, and community-based activities, which are more accessible for individuals living in rural areas.

Despite these promising approaches, this review also revealed an important gap. Most interventions were health-professional-driven programs, and relatively few studies explored resident-driven or community-initiated exercise programs. Given that long-term cancer survivorship often occurs outside healthcare settings, sustainable physical activity promotion may require stronger engagement of community members and local resources [28,29].

Previous community health studies have suggested that community participation and resident-driven initiatives can enhance sustainability and adherence in health promotion programs [30,31]. Therefore, incorporating community participation and local leadership into exercise programs may help improve the long-term effectiveness of survivorship interventions in rural settings.

Strengths of the Study

This study has several strengths. First, it specifically focused on breast cancer survivors living in rural or non-urban settings, a population that has often been underrepresented in cancer survivorship research. By synthesizing evidence across multiple intervention types, this review provides a comprehensive overview of physical activity interventions that can be delivered outside major medical centers. Second, this review included both clinical outcomes and implementation-related factors, such as adherence, feasibility, and delivery methods. This approach provides practical insights into how exercise programs can be implemented in geographically dispersed communities. Third, the review highlights the potential of remote and community-based interventions, including tele-exercise and telephone-based programs, which may help reduce access barriers and promote equitable survivorship care.

Limitations

Several limitations should be considered when interpreting the findings of this review. First, the number of included studies was relatively small, and the sample sizes varied considerably across studies. This limited the ability to perform a meta-analysis and restricted the conclusions to a narrative synthesis of available evidence. Second, there was substantial heterogeneity among the included studies in intervention design, outcome measures, and follow-up duration. This variability made direct comparisons between studies difficult. Third, many interventions relied primarily on healthcare professionals to deliver exercise programs. While these approaches may be effective in controlled research settings, their long-term sustainability in rural communities may be limited without stronger community engagement. Finally, the included studies rarely evaluated resident-driven physical activity initiatives, underscoring the need for further research into community-based models that empower residents to lead exercise programs for cancer survivors.

## Conclusions

Physical activity interventions appear to improve physical activity participation, functional performance, and quality of life among breast cancer survivors living in rural areas. Remote delivery approaches, including telephone-based counseling and tele-exercise programs, may be particularly useful for overcoming geographic barriers to exercise participation. However, most existing programs remain healthcare-professional-driven, and evidence regarding resident-driven exercise initiatives is limited. Future survivorship programs may benefit from stronger collaboration between medical professionals and local communities to develop sustainable exercise opportunities for cancer survivors. Promoting partnerships between healthcare providers and residents may help establish accessible, community-based physical activity programs that support long-term health and quality of life for cancer survivors.

## Appendices

Detailed search strategy

PubMed/MEDLINE

("Breast Neoplasms"[MeSH] OR "breast cancer" OR "breast cancer survivors") AND ("Motor Activity"[MeSH] OR exercise OR "physical activity" OR rehabilitation OR "exercise therapy") AND ("Rural Population"[MeSH] OR rural OR non-urban OR remote OR underserved)

Embase

('breast cancer'/exp OR 'breast cancer survivor' OR 'breast cancer') AND ('physical activity'/exp OR exercise OR rehabilitation OR 'exercise therapy') AND ('rural population'/exp OR rural OR non-urban OR remote OR underserved)

Web of Science

TS=(("breast cancer" OR "breast cancer survivors") AND ("physical activity" OR exercise OR rehabilitation OR "exercise therapy") AND (rural OR non-urban OR remote OR underserved))
